# Using a zero-inflated model to assess gene flow risk and coexistence of *Brassica napus* L. and *Brassica rapa* L. on a field scale in Taiwan

**DOI:** 10.1186/s40529-020-00294-2

**Published:** 2020-05-20

**Authors:** Yuan-Chih Su, Po-Shung Wang, Jhih-Ling Yang, Hong Hong, Tzu-Kai Lin, Yuan-Kai Tu, Bo-Jein Kuo

**Affiliations:** 1 Department of Agronomy, National Chung Hsing University, No. 145 Xingda Road, South District, Taichung City, 40227 Taiwan (R.O.C.); 2 Division of Crop Science, Taiwan Agricultural Research Institute, No. 189, Zhongzheng Road, Wufeng District, Taichung City, 41362 Taiwan (R.O.C.); 3 Division of Biotechnology, Taiwan Agricultural Research Institute, No. 189, Zhongzheng Road, Wufeng District, Taichung City, 41362 Taiwan (R.O.C.); 4 Innovation and Development Center of Sustainable Agriculture (IDCSA), National Chung Hsing University, No. 145 Xingda Road, South District, Taichung City, 40227 Taiwan (R.O.C.)

**Keywords:** Zero-inflated model, Isolation distance, Gene flow, Rapeseed, Genetically modified crop, Coexistence

## Abstract

**Background:**

The cropping area of genetically modified (GM) crops has constantly increased since 1996. However, currently, cultivating GM crops is associated with many concerns. Transgenes are transferred to non-GM crops through pollen-mediated gene flow, which causes environmental problems such as superweeds and introgressive hybridization. Rapeseed (*Brassica napus* L.), which has many GM varieties, is one of the most crucial oil crops in the world. Hybridization between *Brassica* species occurs spontaneously. *B. rapa* grows in fields as a weed and is cultivated as a crop for various purposes. Both *B. rapa* weeds and crops participate in gene flow among rapeseed. Therefore, gene flow risk and the coexistence of these two species should be studied.

**Results:**

In this study, field experiments were conducted at two sites for 4 years to evaluate gene flow risk. In addition, zero-inflated models were used to address the problem of excess zero values and data overdispersion. The difference in the number of cross-pollination (CP) events was nonsignificant between upwind and downwind plots. The CP rate decreased as the distance increased. The average CP rates at distances of 0.35 and 12.95 m were 2.78% and 0.028%, respectively. In our results, zero-inflated negative binomial models were comprehensively superior to zero-inflated Poisson models. The models predicted isolation distances of approximately 1.36 and 0.43 m for the 0.9% and 3% threshold labeling levels, respectively.

**Conclusions:**

Cultivating GM crops is prohibited in Taiwan; however, the study results can provide a reference for the assessment of gene flow risk and the coexistence of these two species in Asian countries establishing policies for GM crops.

## Background

The acreage of global genetically modified (GM) crops has increased to approximately 191,700,000 ha since 1996 (ISAAA 2018). The most common GM crops are maize, soybean, cotton, and canola. GM crops can be classified according to herbicide tolerance (HT), insect resistance, stacked traits, virus tolerance, and other traits; HT GM crops are the most common. Because of the increasing world population, GM crops are considered a solution for ensuring the food security of the world population (Taheri et al. 2017). For example, HT GM crops can provide convenient weed control at a relatively low price (Brookes and Barfoot 2016). Although GM crops have benefits, some issues should be considered. GM crop cultivation is associated with several concerns, including biodiversity, economics, agricultural production, and consumer choice (Smyth et al. 2002). GM crops affect non-GM crops through gene flow and cause the contamination of non-GM crops with transgenes. Therefore, many countries have established a threshold of GM content among non-GM products. The strictest threshold is 0.9% in the Regulation (EC) No. 1830/2003 of the European Union (EU). Therefore, the coexistence of GM and non-GM crops is an issue that must be discussed.

Rapeseed (*Brassica napus* L.) is a cross-pollinated crop of the *Brassica* genus that is typically pollinated by insects. Bees (*Apoidea* superfamily) are its main pollinator (Scheffler et al. 1995). Although *B. napus* is typically pollinated by insects, studies have indicated that *B. napus* can be pollinated without insects (Eisikowitch 1981). Research on gene flow between non-GM and GM *B. napus* has been conducted in the past few years (Beckie et al. 2003). There is evidence that pollination occurs between *B. napus* and its related species (Warwick et al. 2003), and the probability of GM genes being transferring to related species should be examined. Gene flow between *B. napus* and *B*. *juncea* L. was evaluated in a previous study (Zhang et al. 2018a). Studies have also reported that spontaneous hybridization is more likely to occur between *B. napus* and *Brassica. rapa* L. than between *B. napus* and other *Brassica* crops (Landbo et al. 1996). A wild *B. rape* population near a *B. napus* field was revealed to have a hybridization rate of 1.1–17.5% (Simard et al. 2006). Furthermore, a study indicated that introgression hybridization may have occurred between *B. napus* and *B. rapa* (Hansen et al. 2001). Hence, the risk of gene flow between *B. napus* and *B. rapa* is relatively higher than that between *B. napus* and other *Brassica* species.

The most common measure used for determining the coexistence of GM and non-GM *B. napus* is isolation distance. Models that fit the relationship between the cross-pollination (CP) rate and isolation distance have been developed in previous studies, and the optimal isolation distance can be derived from the model with the best fit (Funk et al. 2006; Walklate et al. 2004; Weekes et al. 2005). The pollen dispersal model can be divided into empirical and mechanistic models. Because mechanistic models are difficult to set up for insect pollination, the *B. napus* pollen dispersal model is classified as an empirical model (Klein et al. 2006). The variability of data from dispersal experiments is typically great (Bensadoun et al. 2016). Data are overdispersed when the observed variance is higher than the theoretical variance because of the excess zero values in the observed dispersal count data. To fit this type of data, the zero-inflated Poisson (ZIP) distribution is an appropriate method (Bensadoun et al. 2014).

Small farming systems are common in many Asian countries. In Taiwan, fields are small and scattered (Hsu 2014). An average of 0.3 ha of agricultural land is owned by each person among farming families (Council of Agriculture 2017). Gene flow in Asian farm systems has not been thoroughly studied. Therefore, establishing an optimal field design for GM and non-GM crops to coexist would be beneficial for Asian agricultural development. Few studies have assessed the coexistence of *B. napus* and *B. rapa* on a small field scale. In Taiwan, *B. rapa* is cultivated in fields as a green manure, vegetable, or honey plant. Therefore, adjacent fields of *B. rapa* and GM *B. napus* may cause unexpected gene flow between these species. This study provides new insights into gene flow between non-GM *B. rapa* and GM *B. napus* and how the wind direction and distance affect gene flow during a 4-year experiment. Models that fit the CP rate (%) were also developed. This study provides a valuable reference for researchers and growers interested in preventing gene flow in coexisting of non-GM *B. rapa* and GM *B. napus*.

## Materials and methods

### Plant materials

Non-GM *B. napus* “Deza oil No. 18” (AACC, 2n = 38) was used as the pollen donor in this study. This cultivar has recessive genetic male sterility and is double cross variety, and its growth period is approximately 224 days. The pollen recipient plant was the open-pollination variety (Nongxing 80-day) of *B. rapa* (AA, 2n = 20), which is mainly used as a green manure crop in Taiwan. *B. napus* seedlings were treated with vernalization to ensure flowering in Taiwan. *B. napus* seedlings were cooled to 4 °C for at least 30 days. After *B. napus* vernalization, *B. napus* and *B. rapa* seedlings were planted in 128-well plastic trays in a greenhouse. The seedlings were transplanted to a field until the five-leaf stage.

### Experiment design

**Fig. 1:**
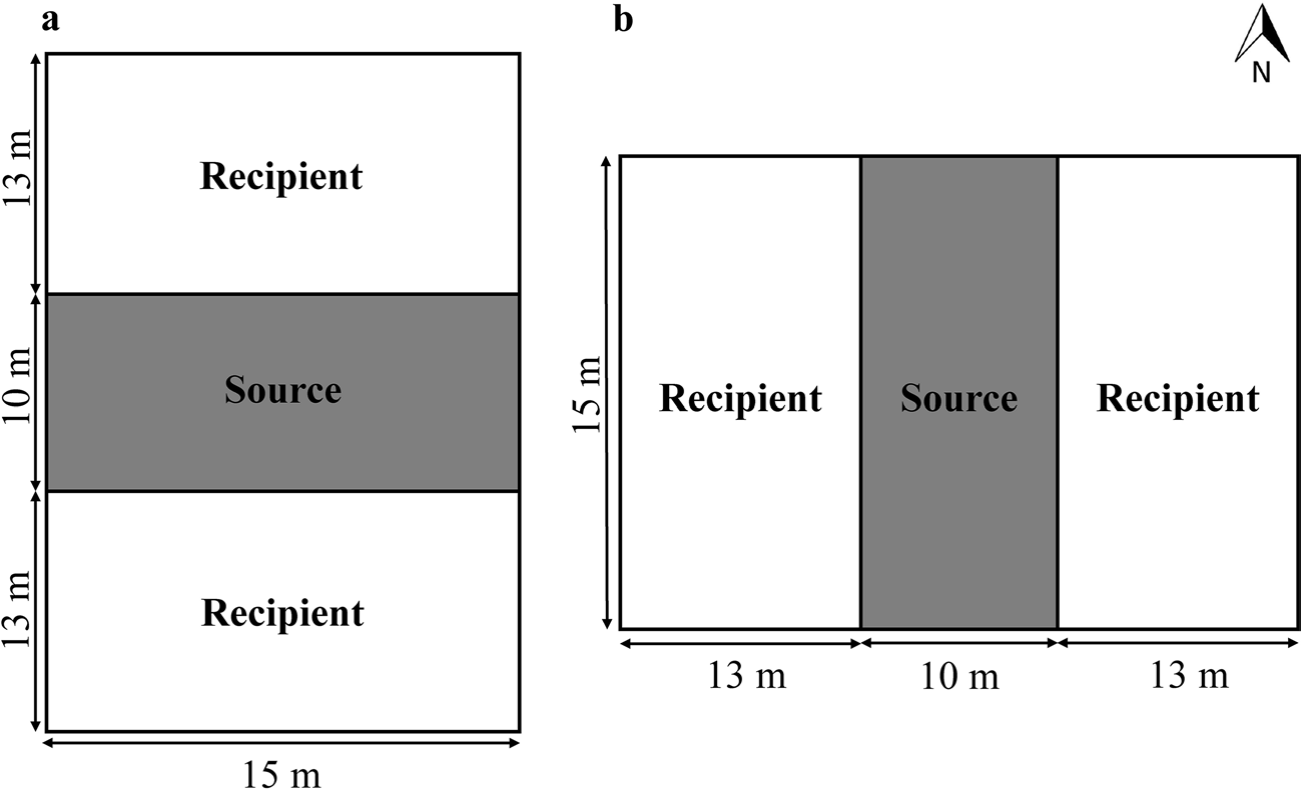
Field design of experiment sites: **a** the Taiwan Agricultural Research Institute and **b** the Agricultural Experiment Station

The pollen dispersal experiments were conducted from the fall of 2013 to the spring of 2017 at Taiwan Agricultural Research Institute (TARI), Council of Agriculture (COA), Executive Yuan (24° 03′ N, 120° 69′ E), and Agricultural Experiment Station (AES), College of Agriculture and Natural Resources, National Chung Hsing University (24° 07′ N, 120° 71′ E). The experiments were replicated eight times, four times for each site. The total area of the two experimental sites was approximately 0.054 ha (36 × 15 m^2^; Fig. 1; Hong et al. 2016; Su 2015; Wang 2017; Yang 2018).

The two pollen recipient plots were located next to the pollen donor plot to simulate adjacent field arrangements in Taiwan (Nieh et al. 2014). The field design of the experiment was established at TARI, where the two recipient plots were located on the north and south sides of the source plot. At the AES site, the two recipient plots were set up on the west and east sides of the source plot. Each experimental field had 12 furrows, and each furrow had two rows. There were 696 and 1776 *B. napus* and *B. rapa* plants in each field, respectively. Blooming was controlled through cutting early flowers to assure flower synchronization.

Meteorological information was recorded by a weather station located at TARI. The daily maximum frequency of the wind direction was taken as the field prevailing wind direction of each day. The proportion of each wind direction during the flowering period was defined as the field prevailing wind direction.

The recipient plants were sampled in two rows of each furrow (except the first and last furrow) at different distances. The sampling distance was in the range of 0.35–12.95 m at 0.7-m intervals. One or two flower stalks were cut for each plant. Mature pods were dried, threshed, and stored for inspecting the hybridization of recipient plants.

### Hybrid progeny screening

**Fig. 2:**
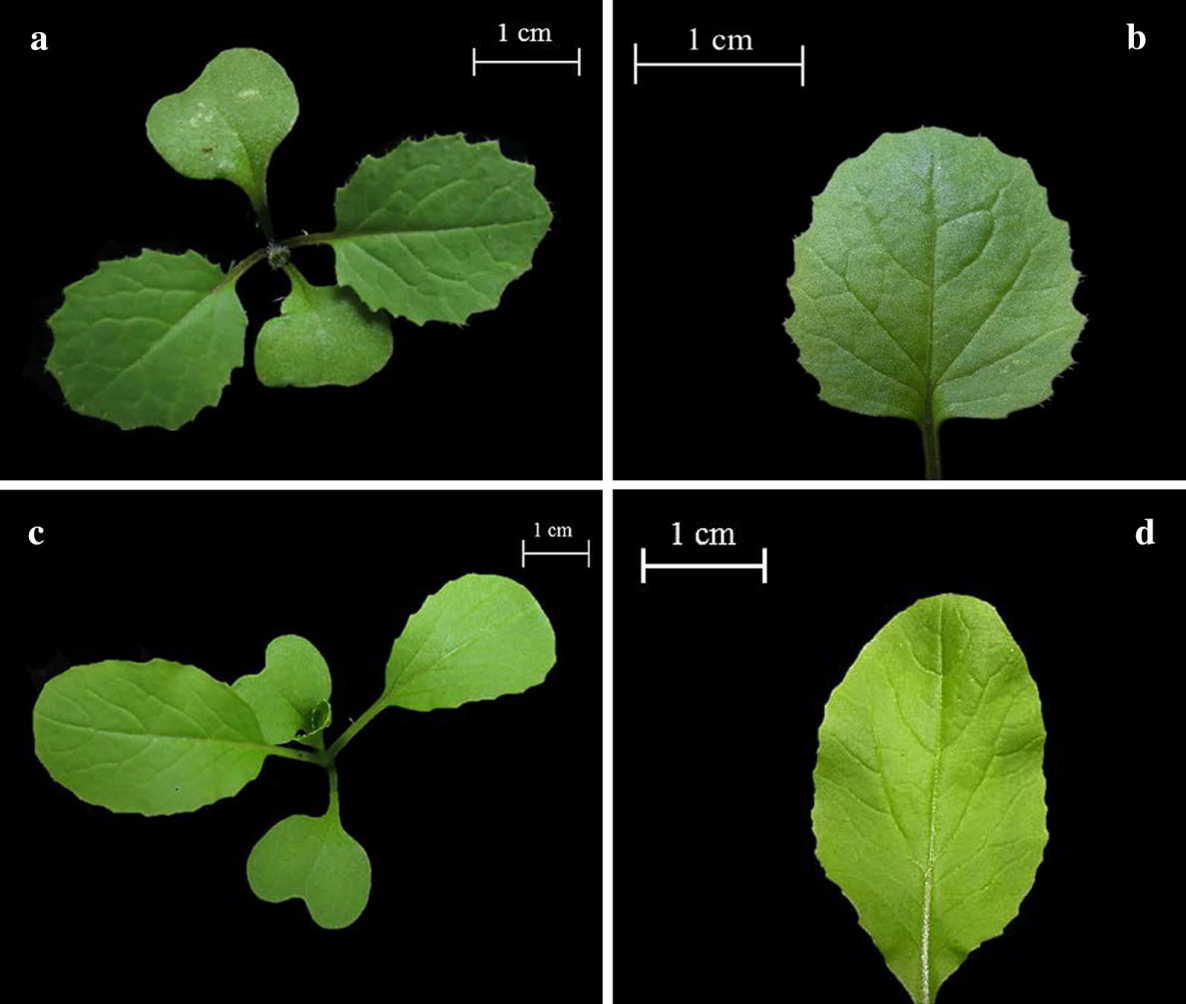
Two-leaf stage progenies. **a** Plant of outcrossing progeny. **b** Leaf shape of outcrossing progeny. **c** Plant of nonoutcrossing progeny. **d** Leaf shape of nonoutcrossing progeny

A previous study discovered that the hybrids of *B. rapa *× *B. napus* could be distinguished from their parents through morphology (Jørgensen and Andersen 1994; Lu et al. 2001; Tu et al. 2020). The morphology characteristics of *B. napus*, *B. rapa* and *B. rapa *× *B. napus* (F1) were described in Tu et al. (2020). The difference between F1 hybrid and parents also showed in the genome size and molecular marker (Tu et al. 2020). In this study, leaf characteristics were used to differentiate between hybrid and nonhybrid progenies at the two-leaf stage. The hybrid leaves were circular, dark green, and displayed a trichome and strong dentation at the margin (Fig. 2a, b). By contrast, the nonhybrid leaves were thin oval shape, light green (Fig. 2c, d).

For each sample, 384 seeds were sowed in plastic trays, and the number of hybrid progenies was counted. The CP rate (%) was calculated by counting the number of outcrossing progenies in each seeding sample, as follows (Eq. 1):

1 $$ {\text{CP }}\left( {\text{\% }} \right) = \frac{{n_{c} }}{N} \times 100\% $$

where *n*_*c*_ is the number of hybrid progenies, and *N* is the total seedling number of the sample. Because of model fitting requirements, the CP rates were transformed into count data by multiplying them by 384 and rounding the value.

### Zero-inflated model

According to previous studies, the CP rate decreases with increasing distance (Beckie et al. 2003; Damgaard and Kjellsson 2005). Therefore, this may result in a relatively large number of zero values in the CP data. Most of models typically demonstrate poor predictive performance when fitted with excess zero values (Rodriguez 2013). The zero-inflated model has been proposed to address the problem of excess zero-count data (Greene 1994; Lambert 1992).

The ZIP model is a model consisting of a fixed zero count and a Poisson distribution. The ZIP model increases the probability of the occurrence of zero values to address excess zero counts. Assume that the probability of zero counts is *π*_*i*_, and the response variable *Y*_*i*_, *i *= 1, 2, 3…, *n*, is a counting variable with a probability density function (pdf; Eq. 2):

2 $$ {\text{P}}\left( {Y_{i} = y_{i} ;\mu_{i} , \pi_{i} } \right) = \left\{ {\begin{array}{*{20}l} {\pi_{i} + \left( {1 - \pi_{i} } \right)e^{{ - \mu_{i} }} , \quad y_{i} = 0} \\ {\left( {1 - \pi_{i} } \right)\frac{{\mu_{i}^{{y_{i} }} }}{{y_{i} !}}e^{{ - \mu_{i} }} , \qquad y_{i} > 0} \\ \end{array} } \right. $$

where *μ*_*i*_ is the parameter of the Poisson distribution. The parameter *μ*_*i*_ satisfies the log link function (Eq. 3). We defined the predictor of *μ*_*i*_ as *Q* × *r(x, y)*. The parameter *Q* and dispersal kernel function *r(x, y)* were introduced in a previous study (Bullock et al. 2017). Dispersal kernel functions include log-sech, exponential power, power law, logistic, 2Dt, gamma, WALD, Weibull, Exponential, log-normal, and Gaussian. Variables *x* and *y* are the two-dimensional coordinates. The parameter *π*_*i*_ is defined as the logit link function (Eq. 4). The predictor for *π*_*i*_ is the same as that for *μ*_*i*_.

3 $$ \mu_{i} = \exp \left( {Q \times r\left( {x,y} \right)} \right) $$

4 $$ \pi_{i} = \frac{{\mu_{i} }}{{1 + \mu_{i} }} $$

Bias may remain in parameter estimation when the ZIP model fits the overdispersed data. Therefore, another zero-inflated model, the zero-inflated negative binomial (ZINB) model, was suggested to solve this problem. The concept of the ZINB model is similar to that of the ZIP model. Because the ZINB model adds a parameter to evaluate the dispersion of data, it is more suitable for overdispersed data. The pdf of the ZINB model is analogous to that of the ZIP model (Eq. 5).

5 $$ {\text{P}}\left( {Y_{i} = y_{i} ; \mu_{i} , \pi_{i} } \right) = \left\{ {\begin{array}{*{20}l} {\pi_{i} + \left( {1 - \pi_{i} } \right) \cdot g\left( {y_{i} } \right), \quad y_{i} = 0} \\ {\left( {1 - \pi_{i} } \right) \cdot g\left( {y_{i} } \right), \qquad y_{i} > 0} \\ \end{array} } \right. $$

6 $$ g\left( {y_{i} } \right) = \frac{{\varGamma \left( {y_{i} + \alpha^{ - 1} } \right)}}{{\varGamma \left( {\alpha^{ - 1} } \right)\varGamma \left( {y_{i} + 1} \right)}}\left( {\frac{1}{{1 + \alpha \mu_{i} }}} \right)^{{\alpha^{ - 1} }} \left( {\frac{{\alpha \mu_{i} }}{{1 + \alpha \mu_{i} }}} \right)^{{y_{i} }} $$

The function *ɡ(y*_*i*_*)* is the pdf of the negative binominal distribution, where Γ is the gamma function, and *α* is the shape parameter. The definitions of *μ*_*i*_ and *π*_*i*_ in the ZINB model are the same as those in the ZIP model (Eqs. 3 and 4).

To apply the two-dimensional function *r*(*x, y*), the distance between individual plants is calculated using Eq. 7. The experimental field is considered a two-dimensional coordinate plane where plant positions are defined by a coordinate point. In Eq. 7, coordinate points (*x, y*) and (*x’, y’*) define the positions of the recipient and donor plants, respectively.

7 $$ {\text{distance}} = \sqrt {\left( {x - x^{\prime}} \right)^{2} - \left( {y - y^{\prime}} \right)^{2} } $$

### Statistical analysis

We expected that wind would not influence the number of CP events. A CP event was defined as the occurrence of CP at a sampling point. We compared the number of CP events in the two recipient plots by using a z-test to evaluate the wind effect. In addition, this study conducted an ANOVA to test the wind effect to the variation of CP rate. Examination of excess zero values was conducted by counting the frequency of zero values among the data and comparing this with the predicted zero frequency of the Poisson distribution. There were excess zero values if the number of zero events was more than expected. Overdispersion was examined based on the assumption of Poisson distribution. If the variance was higher than the mean, then overdispersion may have occurred in the data. In addition, we calculated the deviance by fitting the data with the Poisson distribution, and we computed the ratio of deviance to the degree of freedom (d.f.). A dataset with a ratio of > 1 is considered to be overdispersed (McCullagh and Nelder 1989).

The data of each year and site were combined, and 70% of the total data were randomly selected to train the model. The remaining 30% of the data were the validation dataset. The performance of model fitting was evaluated based on root mean square error (RMSE), adjusted coefficient of determination (adj. R^2^), Akaike’s information criterion (AIC), and Schwarz’s Bayesian information criterion (BIC; Akaike 1974; Schwarz 1978). We selected models with small values of RMSE, AIC, and BIC. A large adj. R^2^ value demonstrated a good model fit. The predictive capability of the model was assessed based on the mean squared prediction error (MSPR). In our study, a model with a small MSPR value was selected as the best model (Jung and Hu 2015). The model selection procedures identified models with a good predictive ability based on the aforementioned criteria recommended for application. The conservative isolation distance at various thresholds was estimated through 500 bootstrapping simulations. The 95th percentile of the distance generated through the simulations was considered the conservative isolation distance. All statistical analyses were performed using SAS 9.4 (SAS Institute Inc., Cary, NC, USA) and R v 3.4.0 (R Development Core Team 2017) software.

## Results

### Wind direction during the flowering period

An overlap of at least 24 days occurred between the donor and recipient plant flowering periods during the eight experiments (Additional file 1: Table S1). In most experiments, the overlap period was longer than 1 month. The AES and TARI sites were located nearby; therefore, we applied meteorological data from the same weather station to both sites. The prevailing wind direction during the flowering period was north (Additional file 2: Table S2).

The relative frequency of northerly winds ranged from 25 to 88%. The two recipient plots in the TARI experiments were assumed to be upwind and downwind plots to evaluate the wind effect on pollination. Because the field arrangement and prevailing wind direction were not parallel, the recipient plots in the AES experiments could not be defined as upwind and downwind plots.

### Distance and wind effects on CP

In the TARI experiments, the CP rates of the upwind and downwind plots were observed separately. The CP rates of both recipient plots of the AES experiments were observed jointly. The average CP rate fluctuated between 0.48% and 5.07% over the shortest distance (0.35 m; Table 1).

**Table 1:** Mean and standard deviation of the observed cross-pollination rate (%)

Distance (m)	CP (%) at TARI Mean ± SD								CP (%) at AES Mean ± SD			
	2013-1		2014-1		2015-1		2016-1					
	Up	Down	Up	Down	Up	Down	Up	Down	2013-2	2014-2	2015-2	2016-2
0.35	1.87 ± 2.01	3.26 ± 2.21	5.07 ± 4.98	3.42 ± 2.75	4.50 ± 3.33	3.43 ± 2.87	2.21 ± 1.83	1.58 ± 0.91	2.36 ± 2.21	0.48 ± 0.68	3.16 ± 4.03	1.98 ± 2.26
1.05	0.47 ± 0.51	1.17 ± 1.06	1.11 ± 1.56	1.03 ± 1.53	1.35 ± 1.76	1.88 ± 2.99	0.70 ± 0.44	0.51 ± 0.59	0.96 ± 0.81	0.17 ± 0.34	0.92 ± 1.02	1.08 ± 1.50
1.75	0.26 ± 0.36	0.30 ± 0.46	0.34 ± 0.30	0.71 ± 1.17	0.31 ± 0.40	0.85 ± .123	0.11 ± 0.25	0.81 ± 0.91	0.39 ± 0.57	0.15 ± 0.26	0.56 ± 0.97	0.38 ± 0.67
2.45	0.31 ± 0.68	0.20 ± 0.23	0.88 ± 1.51	0.22 ± 0.36	0.44 ± 0.73	0.22 ± 0.51	0.18 ± 0.31	0.41 ± 0.50	0.23 ± 0.36	0.00 ± 0.00	0.16 ± 0.25	0.28 ± 0.41
3.15	0.34 ± 0.38	0.34 ± 0.32	0.15 ± 0.21	0.47 ± 1.08	0.09 ± 0.15	0.11 ± 0.34	0.19 ± 0.28	0.15 ± 0.20	0.09 ± 0.17	0.08 ± 0.37	0.29 ± 0.38	0.12 ± 0.29
3.85	0.48 ± 0.68	0.25 ± 0.23	0.06 ± 0.20	0.31 ± 0.44	0.03 ± 0.10	0.11 ± 0.24	0.07 ± 0.14	0.15 ± 0.27	0.10 ± 0.20	0.04 ± 0.14	0.12 ± 0.26	0.05 ± 0.12
4.55	0.10 ± 0.22	0.02 ± 0.08	0.80 ± 1.74	0.05 ± 0.17	0.76 ± 1.76	0.51 ± 0.89	0.06 ± 0.12	0.19 ± 0.27	0.13 ± 0.24	0.00 ± 0.00	0.07 ± 0.18	0.06 ± 0.14
5.25	0.03 ± 0.08	0.07 ± 0.16	0.17 ± 0.26	0.14 ± 0.29	0.00 ± 0.00	0.24 ± 0.51	0.00 ± 0.00	0.00 ± 0.00	0.05 ± 0.11	0.00 ± 0.00	0.00 ± 0.00	0.03 ± 0.08
5.95	0.10 ± 0.21	0.03 ± 0.11	0.17 ± 0.44	0.06 ± 0.20	0.13 ± 0.32	0.13 ± 0.30	0.07 ± 0.15	0.03 ± 0.10	0.08 ± 0.18	0.02 ± 0.07	0.04 ± 0.13	0.06 ± 0.13
6.65	0.05 ± 0.17	0.03 ± 0.08	0.06 ± 0.17	0.11 ± 0.19	0.05 ± 0.16	0.14 ± 0.23	0.03 ± 0.09	0.12 ± 0.29	0.13 ± 0.20	0.00 ± 0.00	0.05 ± 0.18	0.04 ± 0.11
7.35	0.04 ± 0.13	0.03 ± 0.10	0.05 ± 0.16	0.06 ± 0.13	0.05 ± 0.16	0.06 ± 0.13	0.11 ± 0.27	0.06 ± 0.20	0.00 ± 0.00	0.00 ± 0.00	0.05 ± 0.12	0.03 ± 0.10
8.05	0.04 ± 0.14	0.00 ± 0.00	0.10 ± 0.16	0.13 ± 0.22	0.22 ± 0.36	0.00 ± 0.00	0.04 ± 0.12	0.03 ± 0.09	0.07 ± 0.22	0.00 ± 0.00	0.06 ± 0.19	0.03 ± 0.10
8.75	0.00 ± 0.00	0.00 ± 0.00	0.03 ± 0.09	0.03 ± 0.09	0.00 ± 0.00	0.00 ± 0.00	0.09 ± 0.18	0.00 ± 0.00	0.00 ± 0.00	0.00 ± 0.00	0.03 ± 0.10	0.04 ± 0.16
9.45	0.07 ± 0.16	0.00 ± 0.00	0.03 ± 0.09	0.06 ± 0.13	0.62 ± 1.94	0.06 ± 0.18	0.03 ± 0.08	0.00 ± 0.00	0.03 ± 0.09	0.01 ± 0.06	0.01 ± 0.06	0.01 ± 0.06
10.15	0.00 ± 0.00	0.00 ± 0.00	0.08 ± 0.09	0.03 ± 0.08	0.10 ± 0.30	0.07 ± 0.22	0.03 ± 0.09	0.00 ± 0.00	0.02 ± 0.07	0.00 ± 0.00	0.04 ± 0.13	0.00 ± 0.00
10.85	0.00 ± 0.00	0.00 ± 0.00	0.00 ± 0.17	0.19 ± 0.34	0.08 ± 0.24	0.00 ± 0.00	0.00 ± 0.00	0.03 ± 0.10	0.00 ± 0.00	0.00 ± 0.00	0.00 ± 0.00	0.00 ± 0.00
11.55	0.07 ± 0.21	0.10 ± 0.23	0.00 ± 0.00	0.00 ± 0.00	0.04 ± 0.12	0.00 ± 0.00	0.00 ± 0.00	0.00 ± 0.00	0.01 ± 0.06	0.03 ± 0.15	0.01 ± 0.06	0.00 ± 0.00
12.25	0.04 ± 0.12	0.00 ± 0.00	0.00 ± 0.00	0.00 ± 0.00	0.06 ± 0.13	0.07 ± 0.22	0.00 ± 0.00	0.00 ± 0.00	0.03 ± 0.08	0.00 ± 0.00	0.00 ± 0.00	0.06 ± 0.19
12.95	0.00 ± 0.00	0.04 ± 0.11	0.00 ± 0.00	0.00 ± 0.00	0.07 ± 0.22	0.11 ± 0.27	0.00 ± 0.00	0.04 ± 0.12	0.04 ± 0.14	0.00 ± 0.00	0.02 ± 0.09	0.02 ± 0.10

CP: cross-pollinated rate; SD: standard deviation; Up: upwind side; Down: downwind side

The maximum and minimum CP rates over 0.35 m were 13.75% and 0%, respectively, in the TARI experiments. The mean CP rate decreased rapidly with increasing distance and was less than 1% at 1.75 m. The CP rate was relatively stable at distances exceeding 5.25 m. Some CP events were still observed at the maximum distance in most experiments. The mean CP rate in the upwind plots was higher than that in the downwind plots at the minimum distance. The standard deviation of the CP rate at 0.35 m was also higher in upwind plots, except for in the 2013-1 experiment. The z-values of experiments 2013-1, 2014-1, 2015-1, and 2016-1 were − 0.7133, − 0.225, 0, and − 0.724, respectively (Additional file 3: Table S3). Based on the z-test results, wind effects on these four experiments were nonsignificant (all p > 0.05). In addition, we combined the data and calculated the z-values. The overall z-value was − 0.8208, and the wind effect remained nonsignificant. The result of ANOVA showed that the wind direction do not have an effect on the variation of CP rate. (Additional file 4: Table S4).

### Testing of excess zeros and overdispersion

In our study, a zero event was defined as an event with a CP rate of 0%. The proportion of zero events among each experiment was 74%, 75%, 71%, 90%, 75%, 77%, 76%, and 79% (Additional file 5: Table S5). The expected proportion of zero events was calculated with the pdf of the Poisson distribution and compared with the observed proportion of zero events. All expected proportions of zero events were smaller than the observed proportions. Therefore, all experimental data had the problem of excess zeros.

The mean and variance of the CP progeny numbers were calculated and compared to roughly check data overdispersion. In each experiment, the variance of the CP progeny number was larger than its mean (Additional file 6: Table S6). Thus, data from the eight experiments may have been overdispersed. Furthermore, all ratios of deviance to d.f. were larger than 1, except for experiment 2014-2. Both approaches indicated that the experimental data were over dispersed.

### Model fitting

Given the absence of overdispersion, the data for model training and validation excluded the data of experiment 2014-2. The remaining data were divided into training and validation datasets, which contained 70% and 30% of the total data, respectively. This study applied the ZIP and ZINB models with dispersal kernel functions to fit the excess zeros and overdispersed data. The ZIP and ZINB models were fitted with the training dataset and were evaluated separately.

According to the criteria, the ZIP model with logistic (ZIP-logistic), 2Dt (ZIP-2Dt), and Weibull (ZIP-Weibull) dispersal kernel functions were the three preferred candidate models (Table 2). All RMSE values of these models were 0.01043. The ZIP-logistic and ZIP-2Dt models were identified as the best models based on the adj. R^2^ criterion (both adj. R^2^ = 0.01097). AIC and BIC also indicated that ZIP-logistic and ZIP-2Dt were the best models (AIC = − 16,978; BIC = − 16,969). The adj. R^2^, AIC, and BIC values of the ZIP-Weibull model were 0.01064, −16,977, and −16,968, respectively. Based on the adj. R^2^ criterion, we selected ZINB-Weibull, ZINB-exponential power, and ZINB-log-sech as the preferred candidate models. All RMSE values of these models were 0.00823. AIC and BIC also identified these models as the best among the ZINB models. The adj. R^2^, AIC, and BIC values of ZINB-Weibull, which is the optimal ZINB model, were 0.38947, −17,860, and −17,853, respectively. The ZINB models were superior to the ZIP models. Even the ZINB model with the worst fitting criterion values performed better than did the ZIP-Weibull model. Consequently, the candidate ZINB models were chosen for the validation procedure.

**Table 2:** Fitting criteria of the ZIP and ZINB models with the training dataset

Dispersal kernel function	ZIP model				ZINB model			
	RMSE	Adj. R^2^	AIC	BIC	RMSE	Adj. R^2^	AIC	BIC
Log-sech	0.01048	0.00286	− 16,963	− 16,954	0.00823	0.38911	− 17,859	− 17,852
Exponential power	0.01049	− 0.001	− 16,956	− 16,947	0.00823	0.38925	− 17,859	− 17,852
Power law	0.01044	0.01014	− 16,976	− 16,967	0.00826	0.38495	− 17,846	− 17,839
Logistic	0.01043	0.01097	− 16,978	− 16,969	0.00826	0.38517	− 17,847	− 17,840
2Dt	0.01043	0.01097	− 16,978	− 16,969	0.00824	0.38769	− 17,855	− 17,847
Gamma	0.01,064	− 0.0292	− 16904	− 16,895	0.00823	0.38884	− 17,858	− 17,851
WALD	0.01044	0.00994	− 16,976	− 16,967	0.00825	0.38542	− 17,848	− 17,841
Weibull	0.01043	0.01064	− 16,977	− 16,968	0.00823	0.38947	− 17,860	− 17,853
Neg. Exponential	0.01044	0.00989	− 16,977	− 16,966	0.00828	0.38183	− 17,838	− 17,829
Log-normal	0.01,044	0.00954	− 16,975	− 16,966	0.00824	0.38717	− 17,853	− 17,846
Gaussian	0.01044	0.00971	− 16,977	− 16,965	0.00826	0.38444	− 17,846	− 17,837

RMSE: root mean square error; adj. R^2^: adjusted coefficient of determination; AIC: Akaike’s information criterion; BIC: Schwarz’s Bayesian information criterion; MSPR: mean squared prediction error

### Model validation and isolation distance recommendation

In accordance with the MSPR, the ZINB-log-sech, ZINB-exponential power, ZINB-gamma, and ZINB-Weibull models had a good predictive ability in the new dataset. These models had small MSPR values of 0.000068767, 0.000068742, 0.000068764, and 0.000068764, respectively (Table 3). The MSPR values of these four models were similar. Based on the best fit, the ZINB-exponential power and ZINB-Weibull models were selected as the final models. The predicted CP rates of the ZINB-exponential power and ZINB-Weibull models were compared with the observed CP rate. The predicted CP rates were higher than the observed CP rates at distances of 0.35, 1.05, and 1.75 m (Table 4). At distances of 2.45, 3.15, 3.85, and 4.55 m, both models underestimated the CP rate. The predicted CP rate varied little and was overestimated at distances larger than 5.25 m.

**Table 3:** Predicting criteria of the ZIP and ZINB models with the validation dataset

Dispersal kernel function	ZINB model				
	RMSE	Adj. R^2^	AIC	BIC	MSPR
Log-sech	0.0083	0.34657	− 7650.3	− 7644.9	0.000068767
Exponential power	0.0083	0.34681	− 7650.5	− 7645.2	0.000068742
Power law	0.00833	0.34179	− 7644.4	− 7639.1	0.000069270
Logistic	0.00831	0.34446	− 7647.7	− 7642.3	0.000068989
2Dt	0.0083	0.34595	− 7649.5	− 7644.1	0.000068832
Gamma	0.0083	0.3466	− 7650.3	− 7644.9	0.000068764
WALD	0.00831	0.34404	− 7647.2	− 7641.8	0.000069033
Weibull	0.0083	0.3466	− 7650.3	− 7644.9	0.000068764
Neg. Exponential	0.00836	0.33831	− 7641.2	− 7633.8	0.000069723
Log-normal	0.0083	0.3456	− 7649.1	− 7643.7	0.000068869
Gaussian	0.00832	0.34363	− 7647.7	− 7640.3	0.000069163

RMSE: root mean square error; adj. R^2^: adjusted coefficient of determination; AIC: Akaike’s information criterion; BIC: Schwarz’s Bayesian information criterion; MSPR: mean squared prediction error

**Table 4:** Actual and predicted cross-pollination rate (%) of the ZINB models by distance

Distance (m)	Average CP (%)^a^	Predicted CP (%)	
		ZINB-Exponential power model	ZINB-Weibull model
0.35	2.8025	3.0087	2.9728
1.05	0.9989	1.1185	1.1617
1.75	0.3321	0.4916	0.4771
2.45	0.4401	0.2614	0.2541
3.15	0.2085	0.1772	0.1765
3.85	0.1497	0.1461	0.1475
4.55	0.3062	0.1351	0.1365
5.25	0.0631	0.1315	0.1323
5.95	0.0518	0.1305	0.1309
6.65	0.078	0.1303	0.1304
7.35	0.0358	0.1302	0.1303
8.05	0.0503	0.1302	0.1302
8.75	0.0256	0.1302	0.1302
9.45	0.0294	0.1302	0.1302
10.15	0.0398	0.1302	0.1302
10.85	0.0256	0.1302	0.1302
11.55	0.0167	0.1302	0.1302
12.25	0	0.1302	0.1302
12.95	0.0308	0.1302	0.1302

CP: cross-pollinated rate

^a^Average cross-pollinated rate of the validation dataset

The thresholds of cross-pollination rates for recommendation were 3%, 1%, and 0.9%, with reference to regulations in Taiwan, Australia, and the EU, respectively. The recommended distance of each threshold was approached in both models (Table 5). For the 3% threshold, 0.425 and 0.431 m were the distances recommended by the ZINB-exponential power and ZINB-Weibull models, respectively. A distance of approximately 1.35 m was recommended to avoid exceeding the 0.9% threshold.

**Table 5:** Isolation distance (m) evaluated by both zero-inflated negative binomial (ZINB)-exponential power and ZINB-Weibull models under threshold values 3%, 1%, and 0.9%, respectively

Threshold (%)	Model	Isolation distance (m)
3	ZINB-Exponential power	0.425
	ZINB-Weibull	0.431
1	ZINB-Exponential power	1.27
	ZINB-Weibull	1.25
0.9	ZINB-Exponential power	1.36
	ZINB-Weibull	1.34

## Discussion

Estimating the CP rate involved setting targets to develop strategies to eliminate hybridization as part of the hybridization risk assessment between GM *B. napus* and *B. rapa* (Wilkinson et al. 2003). According to a study of gene flow between GM and non-GM *B. napus*, the mean CP rates at 2 and 16 m were 2.33% and 0.46%, respectively (Zhang et al. 2018a).

In other studies, the average CP rates observed at 0.5, 1, and 15 m were 2.50%, 1.28%, and 0.13%, respectively (Zhao et al. 2013). The mean CP rates of 2.88% and 1.02% at 0.35 and 1.05 m, respectively, in our study were similar to those in previous studies. However, the mean CP rate of 0.45% and 0.030% at 1.75 and 12.95 m, respectively, in our study were lower than those in previous studies. Given the pollen competition between species and the plant density, the relatively low CP rate was predictable. The spontaneous hybridization rate between GM *B. napus* and *B. rapa* was 0.196% when the two species were planted in adjacent rows (Xiao et al. 2009). A hybridization rate of 1.46% was observed in a wild *B. rapa* population within 30 m of *B. napus* fields in the United Kingdom (Wilkinson et al. 2003). For *B. rapa* interplantation with a *B. napus* field, the hybridization rate was approximately 7% (Warwick et al. 2003). The results of gene flow may differ under particular conditions. In this study, the results reflected gene flow between two small adjacent fields, a typical field arrangement in Asian countries. According to the average CP rate in our experiments, *B. rapa* plants within 1.05 m contained approximately 1.8% of hybrid progenies. Those hybrids may result in immediate harvest loss for a farmer. Furthermore, hybrids containing a transgene may develop into a volunteer population. The volunteer population with the transgene may become transgene donors or herbicide-resistant weeds and cause economic loss in the future. A volunteer population with a transgene may affect the agricultural ecosystem. Consequently, the coexistence of these two species and evaluation of long-term effects on the environment are necessary in Asian countries.

*Brassica napus* and *B. rapa* are pollinated by insects. Several studies have indicated that the wind direction does not affect the gene flow of *B. napus* (Funk et al. 2006; Rieger et al. 2002). To evaluate the wind effect, the prevailing wind direction was recorded in the TARI field, and the recipient plots were established on the upwind and downwind sides of the donor plot. For each experiment at TARI, the proportion of CP events in the two recipient plots was nonsignificantly different. Even after combining data from the four experiments, the proportion of CP events between the upwind and downwind plots was not considerably different. This indicated that wind did not influence gene flow. Another study posited that wind only affects gene flow and contributes to pollination when insect pollinators are scarce (Hayter and Cresswell 2006). A study investigated wind pollination without insects by using nets (Zhang et al. 2018b). Therefore, the contribution of pollination to *B. napus* gene flow may depend on the abundance of insects. Insect pollinators were sufficiently abundant for pollination in the experimental fields; thus, the wind effect was minor in this study.

In a previous study, the ZIP model was introduced to fit the corn CP rate data (Bensadoun et al. 2014). The number of cross-pollinated progenies was assumed to follow a Poisson distribution. However, the CP data typically presented excess zeros and overdispersion; thus, the CP data were assumed to follow a ZIP distribution. In the present study, the test results for excess zeros and overdispersion indicated that our experimental data contained excess zeros and overdispersion, except for experiment 2014-2. The CP rate for short distances was lower in the 2014-2 experiment than in the other experiments, and overdispersion was not present in the 2014-2 experimental data. Due to data characteristics, we used the ZIP and ZINB models to estimate the CP rate. The experimental data were combined, with the exclusion of the 2014-2 experimental data. According to all criteria, the ZINB model was superior to the ZIP model, and the ZINB model was more appropriate for handling count data in excess zeros and overdispersion (Zulkifli et al. 2011). The ZINB-exponential power and ZINB-Weibull models were the two best models for fitting the data. The adj. R^2^ values for the ZINB-exponential power and ZINB-Weibull models were 0.38925 and 0.38947, respectively. The performance of both models was better than the results obtained in a previous study that modeled the CP rate between *B. napus* and its relatives (Zhang et al. 2018a). Model fitting was affected by the variation of the CP rate at short distances. High variation at short distances has also been observed in other studies (Beckie et al. 2003; Damgaard and Kjellsson 2005). The CP rate variation within a few meters of the donor plot may be attributed to insect behavior (Funk et al. 2006). Although the predicted CP rates for the two models were overestimated within 1.75 m, it was acceptable because of the high variation at a short distance. The overall predicted CP rates within 4 m were similar to the average CP rate. The recommended distances were similar for both models. The ZINB-Weibull model provided a relatively conservative isolation distance at strict thresholds. The recommended distance for GM and non-GM *B. napus* at a 0.9% threshold was 0 m (Weekes et al. 2005). For gene flow between *B. napus* and *B. rapa*, 1.35 m was applicable for the 0.9% threshold in our study. The CP process was affected by many factors: differences in experimental scale, species, and model may have led to various results. A method that can integrate all factors is necessary to predict scenarios in future research.

## Conclusion

This study conducted eight experiments at two sites for 4 years to evaluate the risk of gene flow between *B. napus* and *B. rapa* on a small field scale, similar to typical field sizes in Taiwan. The multiple sites and years of these experiments addressed variation in field conditions of each year and site. Therefore, the result was robust to different years and sites. The risk of long-distance gene flow between *B. napus* and *B. rapa* was negligible. However, the risk remains beyond the short distances of adjacent fields. The experiments provided a preliminary gene flow risk assessment between these two species in Taiwan and provided insights for further research and coexistence strategies.

## Supplementary information

**Additional file 1. Table S1.** Flowering periods and overlapping days in all experiments.

**Additional file 2: Table S2**. Wind direction, frequency, and relative frequency in 4-year study period.

**Additional file 3: Table S3.** Results of z-test for wind direction and outcrossing events.

**Additional file 4: Table S4**. ANOVA result of wind effect to the variation of CP rate

**Additional file 5: Table S5.** Percentage of observed zero events and probability of zero events in all experiments.

**Additional file 6: Table S6.** Variance and mean of variable counts in all experiments.

## Data Availability

The data used and analyzed in this study can be provided from the corresponding author for scientific, non-profit purpose.

## Abbreviations

GM: Genetically modified
CP: Cross-pollination
HT: Herbicide tolerance
EU: European Union
ZIP: Zero-inflated Poisson
TARI: Taiwan Agricultural Research Institute
AES: Agricultural Experiment Station
ZINB: Zero-inflated negative binomial
RMSE: Root mean square error
adj. R^2^: Adjusted coefficient of determination
AIC: Akaike’s information criterion
BIC: Schwarz’s Bayesian information criterion
MSPR: Mean squared prediction error
