# Supplementary material for: Using a zero-inflated model to assess gene flow risk and coexistence of Brassica napus L. and Brassica rapa L. on a field scale in Taiwan
Source: Bot Stud. 2020 May 20;61:17. doi: 10.1186/s40529-020-00294-2 (PMC7239968; doi:10.1186/s40529-020-00294-2)
Supplement: Supplementary file 1 — Additional file 1. Table S1. Flowering periods and overlapping days in all experiments. [file 40529_2020_294_MOESM1_ESM.docx]

**Table S1 Flowering periods and overlapping days in all experiments**

| **Site** | **Code** | **Variety** | **Flowering periods** | **Overlapping days** |
| --- | --- | --- | --- | --- |
| TARI | 2013-1 | Deza oil No. 18 | Feb 12 / Mar 23 | 24 |
|  |  | Nongxing 80 days | Feb 02 / Mar 08 |  |
|  | 2014-1 | Deza oil No. 18 | Jan 07 / Feb 27 | 32 |
|  |  | Nongxing 80 days | Dec 24 / Feb 08 |  |
|  | 2015-1 | Deza oil No. 18 | Dec 29 / Mar 08 | 35 |
|  |  | Nongxing 80 days | Dec 22 / Feb 02 |  |
|  | 2016-1 | Deza oil No. 18 | Jan 09 / Mar 10 | 43 |
|  |  | Nongxing 80 days | Jan 02 / Feb 21 |  |
| AES | 2013-2 | Deza oil No. 18 | Jan 29 / Mar 08 | 34 |
|  |  | Nongxing 80 days | Jan 20 / Feb 23 |  |
|  | 2014-2 | Deza oil No. 18 | Jan 17 / Mar 18 | 42 |
|  |  | Nongxing 80 days | Jan 06 / Feb 28 |  |
|  | 2015-2 | Deza oil No. 18 | Jan 05 / Mar 18 | 45 |
|  |  | Nongxing 80 days | Dec 29 / Feb 19 |  |
|  | 2016-2 | Deza oil No. 18 | Jan 14 / Mar 13 | 40 |
|  |  | Nongxing 80 days | Jan 07 / Feb 23 |  |
