# Supplementary material for: Using a zero-inflated model to assess gene flow risk and coexistence of Brassica napus L. and Brassica rapa L. on a field scale in Taiwan
Source: Bot Stud. 2020 May 20;61:17. doi: 10.1186/s40529-020-00294-2 (PMC7239968; doi:10.1186/s40529-020-00294-2)
Supplement: Supplementary file 2 — Additional file 2: Table S2. Wind direction, frequency, and relative frequency in 4-year study period. [file 40529_2020_294_MOESM2_ESM.docx]

**Table S2 Wind direction, frequency, and relative frequency in 4-year study period**

| **Year** | **Wind direction** | **Frequency** | **Relative frequency** |
| --- | --- | --- | --- |
| 2013 | N | 40 | 0.8889 |
|  | NNE | 1 | 0.2222 |
|  | ESE | 1 | 0.0222 |
|  | ES | 1 | 0.0222 |
|  | SSW | 1 | 0.0222 |
|  | W | 1 | 0.0222 |
| 2014 | N | 13 | 0.5200 |
|  | NNE | 5 | 0.2000 |
|  | NNW | 2 | 0.0800 |
|  | NE | 1 | 0.0400 |
|  | ESE | 1 | 0.0400 |
|  | WNW | 1 | 0.0400 |
|  | NW | 1 | 0.0400 |
|  | E | 1 | 0.0400 |
| 2015 | N | 11 | 0.5000 |
|  | NNE | 4 | 0.1818 |
|  | NW | 3 | 0.1364 |
|  | NNW | 2 | 0.0909 |
|  | ESE | 1 | 0.0455 |
|  | WSW | 1 | 0.0455 |
| 2016 | N | 6 | 0.2500 |
|  | NNE | 3 | 0.1250 |
|  | NNW | 3 | 0.1250 |
|  | ESE | 3 | 0.1250 |
|  | NW | 2 | 0.0833 |
|  | E | 2 | 0.0833 |
|  | ES | 1 | 0.0417 |
|  | SSE | 1 | 0.0417 |
|  | SW | 1 | 0.0417 |
|  | W | 1 | 0.0417 |
|  | WNW | 1 | 0.0417 |
