# Supplementary material for: Using a zero-inflated model to assess gene flow risk and coexistence of Brassica napus L. and Brassica rapa L. on a field scale in Taiwan
Source: Bot Stud. 2020 May 20;61:17. doi: 10.1186/s40529-020-00294-2 (PMC7239968; doi:10.1186/s40529-020-00294-2)
Supplement: Supplementary file 3 — Additional file 3: Table S3. Results of z-test for wind direction and outcrossing events. [file 40529_2020_294_MOESM3_ESM.docx]

**Table S3 Results of z-test for wind direction and outcrossing events**

| **Code** |  | **Upwind** | **Downwind** | **Z score** |
| --- | --- | --- | --- | --- |
| 2013-1 | Observed ratio | 0.2316 | 0.2632 | -0.7133 |
|  | Expected ratio | 0.2474 | 0.2474 | (*p*=0.4756) |
| 2014-1 | Observed ratio | 0.2895 | 0.3 | -0.225 |
|  | Expected ratio | 0.2947 | 0.2947 | (*p*=0.8220) |
| 2015-1 | Observed ratio | 0.2526 | 0.2526 | 0 |
|  | Expected ratio | 0.2526 | 0.2526 | (*p*=1.0000) |
| 2016-1 | Observed ratio | 0.2211 | 0.2526 | -0.724 |
|  | Expected ratio | 0.2368 | 0.2368 | (*p*=0.4691) |
| Overall | Observed ratio | 0.2487 | 0.2671 | -0.8208 |
|  | Expected ratio | 0.2579 | 0.2579 | (*p*=0.4117) |
| *p*: p value; the significant level is set at 0.05 | | | | |
