# Supplementary material for: Using a zero-inflated model to assess gene flow risk and coexistence of Brassica napus L. and Brassica rapa L. on a field scale in Taiwan
Source: Bot Stud. 2020 May 20;61:17. doi: 10.1186/s40529-020-00294-2 (PMC7239968; doi:10.1186/s40529-020-00294-2)
Supplement: Supplementary file 4 — Additional file 4: Table S4. ANOVA result of wind effect to the variation of CP rate [file 40529_2020_294_MOESM4_ESM.docx]

| Table S4 ANOVA result of wind effect to the variation of CP rate | | | | |
| --- | --- | --- | --- | --- |
| SOV | SS | DF | MS | *p* value |
| Wind | 0.04735 | 1 | 0.04735 | 0.64 |
| Error | 1.16924 | 6 | 0.19487 |  |
| Total | 1.21659 | 7 |  |  |
