# Supplementary material for: Using a zero-inflated model to assess gene flow risk and coexistence of Brassica napus L. and Brassica rapa L. on a field scale in Taiwan
Source: Bot Stud. 2020 May 20;61:17. doi: 10.1186/s40529-020-00294-2 (PMC7239968; doi:10.1186/s40529-020-00294-2)
Supplement: Supplementary file 5 — Additional file 5: Table S5. Percentage of observed zero events and probability of zero events in all experiments. [file 40529_2020_294_MOESM5_ESM.docx]

**Table S5 Percentage of observed zero events and probability of zero events in all experiments**

| **Code** | **Excess zero** | |
| --- | --- | --- |
|  | **Observation** | **Expectation** |
| 2013-1 | 0.7448 | 0.3621 |
| 2013-2 | 0.7526 | 0.3847 |
| 2014-1 | 0.7057 | 0.2003 |
| 2014-2 | 0.9010 | 0.7768 |
| 2015-1 | 0.7500 | 0.1827 |
| 2015-2 | 0.7734 | 0.3259 |
| 2016-1 | 0.7552 | 0.4541 |
| 2016-2 | 0.7865 | 0.4319 |
| Observation: percentage of observed zero events; Expectation: probability of zero event for a Poisson distribution | | |
