# Supplementary material for: Using a zero-inflated model to assess gene flow risk and coexistence of Brassica napus L. and Brassica rapa L. on a field scale in Taiwan
Source: Bot Stud. 2020 May 20;61:17. doi: 10.1186/s40529-020-00294-2 (PMC7239968; doi:10.1186/s40529-020-00294-2)
Supplement: Supplementary file 6 — Additional file 6: Table S6. Variance and mean of variable counts in all experiments. [file 40529_2020_294_MOESM6_ESM.docx]

**Table S6 Variance and mean of variable counts in all experiments**

| **Code** | **Overdispersion** | | | | |
| --- | --- | --- | --- | --- | --- |
|  | **Variance** | **Mean** | **Deviance** | **d.f.** | **Ratio** |
| 2013-1 | 9.8203 | 1.0158 | 681.29 | 378 | 1.8024 |
| 2013-2 | 9.188 | 0.9553 | 621.3 | 378 | 1.6437 |
| 2014-1 | 30.0226 | 1.6079 | 1174.1 | 378 | 3.1061 |
| 2014-2 | 0.965 | 0.2526 | 310.07 | 378 | 0.8203 |
| 2015-1 | 27.4454 | 1.7000 | 1509 | 378 | 3.9921 |
| 2015-2 | 21.3257 | 1.1211 | 819.23 | 378 | 2.1673 |
| 2016-1 | 5.1798 | 0.7895 | 482.81 | 378 | 1.2773 |
| 2016-2 | 9.3066 | 0.8395 | 657.76 | 378 | 1.7401 |
| Deviance, degree of freedom, and their ratio were computed after Poisson model fitting.  Variance: variance of hybrid progeny number; Mean: mean of hybrid progeny number; d.f.: degree of freedom | | | | | |
